# Dysregulation of Mesenchymal Cell Survival Pathways in Severe Fibrotic Lung Disease: The Effect of Nintedanib Therapy

**DOI:** 10.3389/fphar.2019.00532

**Published:** 2019-05-17

**Authors:** Rajesh K. Kasam, Geereddy B. Reddy, Anil G. Jegga, Satish K. Madala

**Affiliations:** ^1^Department of Pediatrics, College of Medicine, University of Cincinnati, Cincinnati, OH, United States; ^2^Division of Pulmonary Medicine, Cincinnati Children’s Hospital Medical Center, Cincinnati, OH, United States; ^3^Department of Biochemistry, National Institute of Nutrition, Hyderabad, India; ^4^Division of Biomedical Informatics, Cincinnati Children’s Hospital Medical Center, Cincinnati, OH, United States

**Keywords:** myofibroblasts, fibrocytes, apoptosis, idiopathic pulmonary fibrosis, nintedanib

## Abstract

Impaired apoptotic clearance of myofibroblasts can result in the continuous expansion of scar tissue during the persistent injury in the lung. However, the molecular and cellular mechanisms underlying the apoptotic clearance of multiple mesenchymal cells including fibrocytes, fibroblasts and myofibroblasts in severe fibrotic lung diseases such as idiopathic pulmonary fibrosis (IPF) remain largely unknown. We analyzed the apoptotic pathways activated in mesenchymal cells of IPF and in a mouse model of TGFα-induced pulmonary fibrosis. We found that fibrocytes and myofibroblasts in fibrotic lung lesions have acquired resistance to Fas-induced apoptosis, and an FDA-approved anti-fibrotic agent, nintedanib, effectively induced apoptotic cell death in both. In support, comparative gene expression analyses suggest that apoptosis-linked gene networks similarly dysregulated in both IPF and a mouse model of TGFα-induced pulmonary fibrosis. TGFα mice treated with nintedanib show increased active caspase 3-positive cells in fibrotic lesions and reduced fibroproliferation and collagen production. Further, the long-term nintedanib therapy attenuated fibrocyte accumulation, collagen deposition, and lung function decline during TGFα-induced pulmonary fibrosis. These results highlight the importance of inhibiting survival pathways and other pro-fibrotic processes in the various types of mesenchymal cells and suggest that the TGFα mouse model is relevant for testing of anti-fibrotic drugs either alone or in combination with nintedanib.

## Introduction

Idiopathic pulmonary fibrosis (IPF) is a fatal fibrotic lung disease associated with aberrant activation of fibroblasts, leading to their excessive proliferation, survival, accumulation, and production of the extracellular matrix (ECM) (Hardie et al., 2009). In the United States alone, the disease affects approximately 200,000 of whom ∼40,000 die each year. The average survival rate is 2–3 years following diagnosis. Thus, the disease claims more lives annually than many types of cancer (Gribbin et al., 2006; Hutchinson et al., 2014).

Two US Food and Drug Administration (FDA)-approved drugs, Ofev (nintedanib) and Esbriet (pirfenidone), are available as new therapies for IPF patients. Although they improve lung function, the mechanisms underlying their effects remain largely unknown (King et al., 2014; Richeldi et al., 2014; Nathan et al., 2017). During active fibrogenesis, fibroblasts proliferate and transdifferentiate into effector myofibroblasts, which secrete ECM components in the distal parenchymal areas of the lung and cause alveolar destruction and impaired gas exchange (Huang and Horowitz, 2014; Freeman et al., 2017). In a typical wound-healing response, myofibroblasts accumulate quickly after injury to restore the barrier function and initiate repair responses at the site of lung injury. Later, apoptosis clears them to prevent the formation of scar tissue (Desmouliere et al., 1995). However, with persistent injury and excessive growth-factor production, resistance to apoptotic clearance develops in myofibroblasts and fibrotic lung lesions continuously expand (Wynn, 2007; Marudamuthu et al., 2015). Understanding the molecular controls of this dysfuction will clear the way for developing effective antifibrotic drugs.

Many types of mesenchymal cells, including fibrocytes and lung-resident fibroblasts, populate fibrotic lesions and prompt their transformation into myofibrobalsts and formation of permanent scar tissue. Myofibroblasts are terminally differentiated, spindle-shaped cells that express α-smooth muscle actin (αSMA) and accumulate in fibrotic lesions as they attain the anti-apoptotic phenotype and relentlessly secrete ECM components (Desmouliere et al., 1995). Recent findings suggest the activation of survival pathways in myofibroblasts during the pathogenesis of pulmonary fibrosis. In particular, myofibroblasts isolated from the lungs of IPF patients exhibit resistance to Fas-mediated apoptosis (Tanaka et al., 2002). Apoptosis regulators, such as X-linked inhibitor of apoptosis protein (XIAP), Bid, Blc-2, and cellular FLICE inhibitory protein (c-FLIP), are dysregulated (Golan-Gerstl et al., 2012; Ajayi et al., 2013; Safaeian et al., 2014), and several growth factors and mechanotransduction pathways have been shown to activate various prosurvival pathways in myofibroblasts (Thannickal and Horowitz, 2006; Horowitz et al., 2012; Nho et al., 2013). However, the precise network of genes that initiates and maintains resistance to apoptosis in mesenchymal cells remains largely undefined. Understanding these survival pathways in the mesenchymal cells that accumulate in fibrotic lesions will hasten the discovery of novel therapeutic targets in IPF (Glasser et al., 2016).

Fibrocytes are bone marrow-derived mesenchymal cells that express the hematopoietic cell-surface marker CD45 and mesenchymal cell-specific markers, such as collagens and vimentin (Pilling et al., 2009; Maharaj et al., 2013; Sontake et al., 2019). During injury and fibrosis, fibrocytes accumulate in distinct pathological areas of the lung (Madala et al., 2014b). Evaluation of IPF biopsies and mouse models of pulmonary fibrosis suggest that they accumulate with myofibroblasts in mature fibrotic lesions (Moore et al., 2005, 2006; Pilling et al., 2007; Moeller et al., 2009; Pilling and Gomer, 2014), and their excessive accumulation augments fibroproliferation and ECM deposition (Kleaveland et al., 2014; Sontake et al., 2015). Fibrocytes and myofibroblasts persist together and cross-talk to maintain mature fibrotic lesions, but we do not know how survival pathways impair their clearance. Elucidating this process is crucial for developing therapeutic strategies that target multiple mesenchymal cells and induce regression of fibrotic lesions.

Nintedanib (BIBF 1120) is an indolinone derivative that binds to the ATP pockets of vascular endothelial growth-factor receptor (VEGFR), platelet-derived growth-factor receptor (PDGFR), and fibroblast growth-factor receptor (FGFR), blocking their tyrosine- mediated kinase activity (Hilberg et al., 2008). Nintedanib has proven effective in reducing the fibrotic burden and lung function decline in patients with IPF (Richeldi et al., 2014). Unfortunately, the mechanism of action remains elusive. It has been shown to attenuate collagen deposition during bleomycin-induced pulmonary fibrosis (Wollin et al., 2014; Li et al., 2017) and reduced collagen levels in a mouse model of rheumatoid arthritis-associated interstitial lung disease (Redente et al., 2018). It has been shown to attenuate fibroblast proliferation, migration, fibroblast-to-myofibroblast transformation, and ECM synthesis *in vitro* (Wollin et al., 2015; Huang et al., 2016). Also, nintedanib shown to induce the autophagy pathway in fibroblasts isolated from the lungs of patients with IPF (Rangarajan et al., 2016). Although fibrocytes and lung-resident myofibroblasts have been shown to accumulate in fibrotic lesions, the mechanisms that cause their accumulation remain unexplored. Understanding the molecular action of nintedanib as an anti-fibrotic therapy is critical to develop more effective treatments that act either alone or in combination with nintedanib to improve IPF patients’ survival.

## Materials and Methods

### Mouse Model of TGFα-Induced Pulmonary Fibrosis and Nintedanib Therapy

The generation of TGFα-overexpressing mice has been described previously (Hardie et al., 2004). Clara cell-specific protein-rtTA^+/–^ (CCSP-rtTA) mice were crossed with heterozygous (TetO)_7_-cmv TGFα mice to produce bitransgenic CCSP/TGFα mice. To induce TGFα expression, the transgenic mice were fed with doxycycline (Dox)-containing chow (62.5 mg/kg) (Madala et al., 2014c). Both male and female gender mice at 10–16 weeks of age were used in all the studies. They were housed under specific pathogen-free conditions and handled in accordance with protocols approved by the Institutional Animal Care and Use Committee of the Cincinnati Children’s Hospital Research Foundation. Nintedanib (Cayman Chemical, Ann Arbor, MI, United States) was prepared in fresh vehicle (0.5% carboxymethylcellulose) every day before treatment. Fibrosis was induced by overexpressing TGFα for 3 weeks, and in the last 5 days, vehicle or nintedanib (60 mg/kg, once a day) was administered by oral gavage as described (Madala et al., 2016b). For chronic intervention study, all groups of mice were started on Dox for the total 7 weeks. At the beginning of week 4 when fibrosis was extensive, control and TGFα mice were treated with either vehicle or nintedanib for the final 4 weeks (Sontake et al., 2017). Non-TGFα expressing mice on Dox treated with vehicle was used as a control group to determine extent of fibrosis in vehicle and pharmacologically treated groups.

### Human and Mouse Lung Primary Mesenchymal Cell Cultures

Human and mouse lung mesenchymal cell cultures were prepared as described (Sontake et al., 2017, 2018). To isolate fibrocytes and lung-resident myofibroblasts, lung mesenchymal cells were harvested and incubated with anti-CD45 microbeads on ice for 15 min (Miltenyi Biotec, Auburn, CA, United States). After washing twice with sterile buffer, cells were loaded onto magnetic columns (Miltenyi Biotec) and eluted with appropriate amounts of sterile buffer in the presence and absence of a magnetic field to separate unbound cells (CD45^–ve^ cells; lung-resident myofibroblasts or those bound to the column (CD45^+ve^ cells; fibrocytes). Purity of mesenchymal cell subsets was determined using flow cytometry (≥96%) (Madala et al., 2014b). Human and mouse mesenchymal cells were cultured in DMEM with 10% FBS and IMDM with 5% FBS media, respectively. Primary cells used in the experiments were between passages 1–5.

### RNA Extraction and Real-Time PCR

Total RNA was prepared from isolated cells and lung tissue using RNeasy Mini Kit (Qiagen Sciences, Valencia, CA, United States) as described (Madala et al., 2012). Complementary DNA was prepared, and real-time PCR was performed using the CFX384 Touch Real-Time PCR detection system and SYBR green super mix (Bio-Rad, Hercules, CA, United States). Target gene transcripts in each sample were normalized to mouse hypoxanthine guanine phosphoribosyl transferase (Hprt) or human beta-actin. Tables 1, 2 lists the real-time primers used in this study.

**TABLE 1:** The list of mouse RT-PCR primers used in the study.

**Gene symbol**	**Forward**	**Reverse**
Fas	aaaccagacttctactgcgattct	gggttccatgttcacacga
Hprt	gcccttgactataatgagtacttcagg	ttcaacttgcgctcatcttagg
Bak	ggaatgcctacgaactcttca	ccagctgatgccactcttaaa
Bax	gtgagcggctgcttgtct	ggtcccgaagtaggagagga
Spp1	cccggtgaaagtgactgatt	ttcttcagaggacacagcattc
Mdk	cgcactggtaaaaccgaact	gaagaagcctcggtgctg
Grem1	gacccacggaagtgacaga	ccctcagctgttggcagtag
Ptgs2	gggagtctggaacattgtgaa	tgtcaatcaaatatgatctggatgt
Col1α	agacatgttcagctttgtggac	gcagctgacttcagggatg
Fn1	cggagagagtgcccctacta	cgatattggtgaatcgcaga

**TABLE 2:** The list of human RT-PCR primers used in the study.

**Gene symbol**	**Forward**	**Reverse**
FAS	gtggacccgctcagtacg	tctagcaacagacgtaagaacca
β-ACTIN	ccaaccgcgagaagatga	ccagaggcgtacagggatag
BCL-2	agtacctgaaccggcacct	gccgtacagttccacaaagg
SPP1	gagggcttggttgtcagc	caattctcatggtagtgagttttcc
MDK	cccaaagcaatgtgagtcc	gggggaaaaagtcagtttatttg
GREM1	caatttcgttaacggagatgact	caagactgtggtacaagctcctaa
PTGS2	ggatctgtggatgcttcgtt	acccacagtgcttgacacag
CDK1	tggatctgaagaaatacttggattcta	caatcccctgtaggatttgg
CDK4	gtgcagtcggtggtacctg	ttcgcttgtgtgggttaaaa
CCNA2	ggtactgaagtccgggaacc	gaagatccttaaggggtgcaa
PLK1	aacgacttcgtgttcgtggt	agggctttcctcctcttgtg

### Western Blot

Mice lung tissue or primary lung-resident myofibroblasts and fibrocytes treated with DMSO or nintedanib were lysed using RIPA lysis buffer supplemented with protease and phosphatase inhibitors (Cell Signaling Technology, Denver, CO, United States). Total protein was quantified using a BCA kit (Thermo Fisher Scientific, Waltham, MA), and an equal amount of protein from a soluble fraction was subjected to SDS-PAGE on a 4–12% gel as described (Singh et al., 2017). The primary antibodies used were Bak (#12105) and Bax (#2772) from Cell Signaling Technology (Denver, CO, United States), and Collagen1α (Santa Cruz Biotechnology, Dallas, TX, United States) and Gapdh (Bethyl Laboratories). Quantification was performed using the volume integration function of the phosphor imager software, Multigage (Fujifilm, Valhalla, NY, United States) as described (Madala et al., 2016a).

### IncuCyte ZOOM Caspase 3/7 Apoptotic Assay

Kinetic estimation of caspase 3/7 activity was performed using the real-time imaging system IncuCyte ZOOM (Essen BioScience, Ann Arbor, MI, United States). Activation of caspase-3/7 in cells undergoing apoptotic death cleaves the caspase-3/7 substrate to produce nuclear green-fluorescence (Caspase-3/7 Green Apoptosis Assay Reagent [Essen Bioscience]). Primary lung-resident myofibroblasts or fibrocytes were prepared from normal or fibrotic lung tissue and cultured in a 12-well plate to 50–60% confluency. After growing overnight in low serum-containing MEM media, they had adapted to low-serum conditions. They were then treated with media containing either Caspase 3/7 Green Apoptosis Assay Reagent at a final concentration of 5 μM/mL or Caspase 3/7 Green Apoptosis Assay Reagent and anti-Fas antibody (BD Biosciences) at a final concentration of 250 ng/mL. Time-lapse fluorescence imaging was performed using the IncuCyte ZOOM system (Essen BioScience); 9 images per well at 20× magnification were collected every 2 h for 24–48 h. The average number of green objects produced by the apoptotic cells were measured using IncuCyte ZOOM software 2015A.

### Immunohistochemistry and Cell Counting

Formalin-fixed lung sections were prepared and immunostained with antibodies against active caspase-3 (#9664, 1:200, Cell Signaling Technology) and Ki-67 (#12202, 1:600, Cell Signaling Technology) as described (Sontake et al., 2015). From each lung section, 5–10 high-magnification (40×) images of subpleural regions were obtained randomly using a Nikon-Ni-E Upright Microscope. Active caspase-3 or Ki-67-positive cells were counted using MetaMorph imaging software (Molecular Devices, Sunnyvale, CA, United States) as described (Sontake et al., 2015).

### Histology, Pleural Thickness Measurement, and Lung Function Test

Lungs were inflated and fixed using 10% buffered formalin and stained with Mason’s trichrome as described previously (Sontake et al., 2017). To measure pleural thickness, five random images (40×) were collected from each mice using a bright field microscope (Leica Microsystems). Pleural thickness was measured using distance measurement function of MetaMorph imaging software (v6.2: Molecular Devices, Sunnyvale, CA, United States) as described previously (Sontake et al., 2017). Lung function tests were performed by a computerized Flexi Vent system (SCIREQ, Montreal, Canada) as described previously (Sontake et al., 2015).

### BrdU Proliferation Assay

Primary lung-resident fibroblast proliferation was assessed using a BrdU Cell Proliferation Assay Kit (Cell Signaling Technology, Denver, CO, United States) as described (Sontake et al., 2017, 2018). Briefly, primary lung-resident fibroblasts were treated with DMSO or nintedanib (0.1, 0.5, and 1 μM) for 24 h then incubated with BrdU labeling solution for another 24 h along with nintedanib or DMSO. The cells were fixed 24 h after BrdU labeling, and immunodetection of BrdU was performed according to the manufacturer’s protocol. Change in proliferation was calculated as fold difference over control by measuring absorbance at 450 nm.

### Computational Analysis

We performed direct comparison of differentially expressed genes (DEGs) linked with apoptosis between IPF and TGFα mice on Dox for 3 weeks. We used a previously published transcriptomic data set (GSE53845) (DePianto et al., 2015) derived from analysis of the lung biopsies of 40 IPF patients and eight healthy controls and available in the National Center for Biotechnology Information (NCBI) Gene Expression Omnibus (GEO) (Barrett et al., 2007). This IPF gene signature was compared with the DEGs from TGFα mice on Dox for 3 weeks (Sontake et al., 2015). The intersecting up- and down-regulated genes between IPF and TGFα mice on Dox for 3 weeks were then subjected to functional enrichment analysis using the ToppFun application of the ToppGene Suite (Chen et al., 2009). For network representation of select significantly enriched biological processes (apoptosis in this case), Cytoscape application (Shannon et al., 2003) was used.

### Flow Cytometry

Total lung cells obtained from lung stromal cultures of control mice and TGFα mice treated with vehicle or nintedanib were used to stain for CD45 as described previously (Madala et al., 2014b). Data were acquired using BD FACSCANTO II (BD Biosciences) and were analyzed using FACSDIVA software (BD Biosciences).

### Statistical Analysis

All data were analyzed using Prism (version 7.02; GraphPad, La Jolla, CA, United States). Student’s *t*-test was used to compare the two experimental groups. One-way ANOVA with Sidak’s multiple comparison was used to compare the various experimental groups, and two-way ANOVA to compare the independent variables between groups. Data were considered statistically significant for *p* values less than 0.05.

## Results

### Nintedanib Induces Apoptotic Clearance of Lung-Resident Myofibroblasts

**FIGURE 1:**
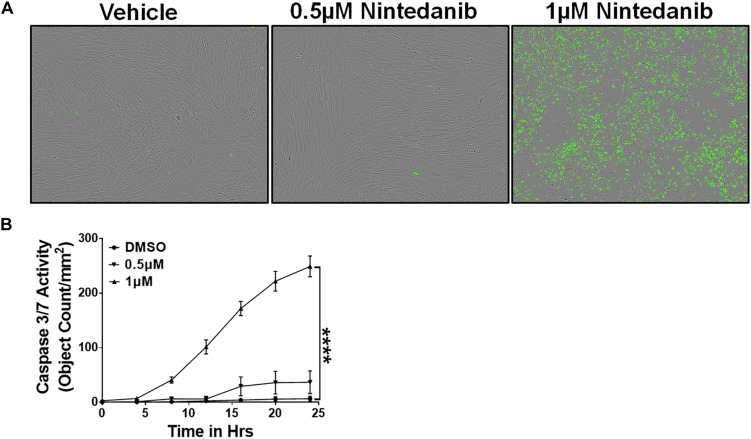
Nintedanib treatment triggers apoptotic clearance of lung-resident myofibroblasts in IPF. Primary lung-resident myofibroblasts (CD45^–^Col1^+^) were isolated from IPF lung fibroblast cultures by negative selection with anti-CD45 magnetic beads. **(A)** Representative images of apoptotic cells (green, active caspase-3/7-positive) in lung-resident myofibroblasts treated with either vehicle or nintedanib (0.5 and 1 μM) for 24 h. **(B)** Quantification of apoptotic cells (green, active caspase-3/7-positive) in lung-resident myofibroblasts treated with either vehicle or nintedanib (0.5 and 1 μM) (*n* = 4). Two-way ANOVA with Sidak’s multiple comparisons test was used to measure significant difference. Data are presented as mean ± SEM (*n* = 4). ****P < 0.00005.

In IPF, the ECM-producing myofibroblasts that accumulate in fibrotic lung lesions develop resistance to apoptosis (Frankel et al., 2006; Thannickal and Horowitz, 2006). Nintedanib was shown to attenuate fibroblast proliferation, migration, and transformation, but its effects on apoptotic clearance had not been explored (Wollin et al., 2015). To assess the effect of nintedanib on apoptotic clearance, lung-resident myofibroblasts of IPF patients were cultured in the presence of a caspase 3/7 substrate conjugated to a green fluorophore and treated with either vehicle or nintedanib (0.5 and 1 μM). We observed a significant increase in cleaved caspase-3 activity and, hence, more apoptotic cells (green) in lung-resident myofibroblasts treated with nintedanib compared to vehicle alone (Figures 1A,B).

**FIGURE 2:**
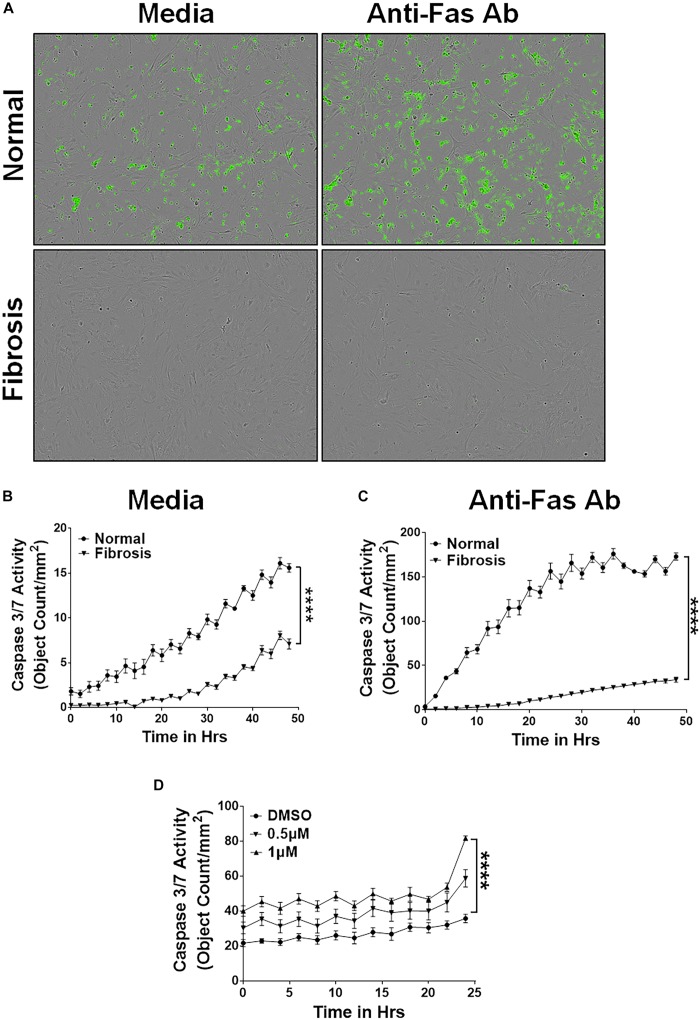
Nintedanib triggers clearance of apoptotic-resistant lung-resident myofibroblasts that accumulate during TGFα-induced pulmonary fibrosis. Primary lung-resident myofibroblasts (CD45^–^Col1^+^) were isolated from lung fibroblast cultures of normal mice and TGFα transgenic mice on Dox for 4 weeks by negative selection with anti-CD45 magnetic beads. **(A)** Representative images of apoptotic cells (green, Caspase-3/7-positive) in lung-resident myofibroblasts cultured with media or FasL (250 ng/ml) for 24 h. **(B)** Quantification of apoptotic cells (green, Caspase-3/7-positive) in lung-resident myofibroblasts isolated from normal and fibrotic lungs of TGFα mice on Dox for 4 weeks and treated with media. **(C)** Quantification of apoptotic cells (green, Caspase-3/7-positive) in lung-resident myofibroblasts isolated from normal and fibrotic lungs of TGFα mice on Dox for 4 weeks and treated with FasL (250 ng/ml) for 48 h. **(D)** Quantification of apoptotic cells (green, Caspase-3/7-positive) in lung-resident myofibroblasts isolated from fibrotic lungs of TGFα mice on Dox for 4 weeks and treated with vehicle or nintedanib (0.5 and 1 μM) for 24 h. Two-way ANOVA with Sidak’s multiple comparisons test was used to measure significant difference. All data are presented as mean ± SEM (*n* = 4). ****P < 0.00005.

In addition, we isolated lung-resident myofibroblasts from the lung cultures of TGFα and control mice on Dox for 4 weeks and treated with media or anti-Fas antibodies. Like the IPF myofibroblasts, resistance to apoptosis was significantly greater in lung-resident myofibroblasts from the TGFα mice than the normal mice (Figures 2A,B). As expected, anti-Fas antibody treatment has resulted in more apoptosis in lung-resident myofibroblasts isolated from normal lungs. Notably, the resistance to apoptotic clearance persisted in lung-resident myofibroblasts isolated from the TGFα mice even in the presence of anti-Fas antibodies (Figures 2A,C). To determine the effect of nintedanib on apoptosis, lung-resident myofibroblasts isolated from the fibrotic lungs of TGFα mice were treated with vehicle or nintedanib, and cleaved caspase-3 activity was measured. The results, shown in Figure 2D demonstrate that nintedanib treatment attenuates apoptosis resistance in lung-resident myofibroblasts of TGFα mice on Dox for 4 weeks.

### Nintedanib Attenuates the Fibrocyte Survival Phenotype

**FIGURE 3:**
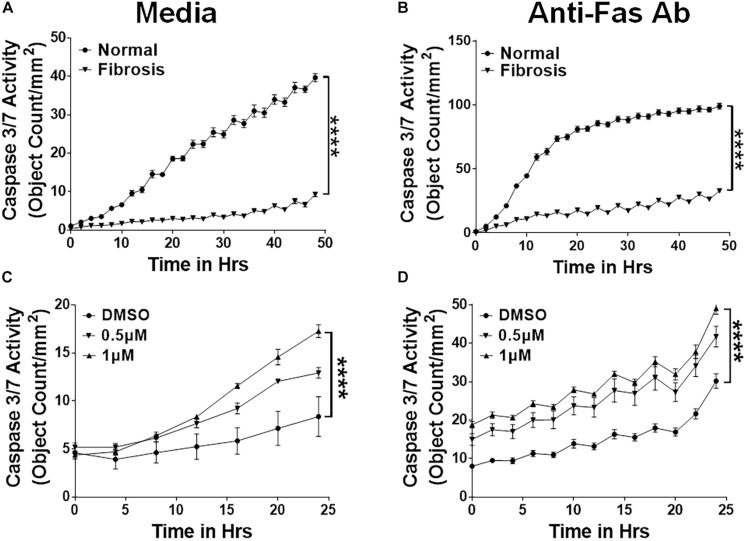
Nintedanib triggers clearance of apoptotic-resistant fibrocytes that accumulate during TGFα-induced pulmonary fibrosis. Primary lung fibrocytes (CD45^+^Col1^+^) were isolated from lung stromal cultures of normal and TGFα transgenic mice on Dox for 4 weeks by positive selection with anti-CD45 magnetic beads. **(A)** Quantification of apoptotic cells (green, active caspase-3/7-positive) in fibrocytes isolated from normal and fibrotic lungs of TGFα mice on Dox for 4 weeks and treated with media (*n* = 4). **(B)** Quantification of apoptotic cells (green, active caspase-3/7-positive) in fibrocytes isolated from normal and fibrotic lungs of TGFα mice on Dox for 4 weeks and treated with FasL (250 ng/ml) for 48 h (*n* = 4). **(C)** Quantification of apoptotic cells (green, active caspase-3/7-positive) in fibrocytes isolated from IPF lungs and treated with either vehicle or nintedanib (0.5 and 1 μM) (*n* = 4). **(D)** Quantification of apoptotic cells (green, active caspase-3/7-positive) in fibrocytes isolated from fibrotic lungs of TGFα mice on Dox for 4 weeks and treated with either vehicle or nintedanib (0.5 and 1 μM) (*n* = 4). Two-way ANOVA with Sidak’s multiple comparisons test was used to measure significant difference. All data are presented as mean ± SEM. ****P < 0.00005.

Others and we have shown that fibrocytes accumulate in fibrotic lung lesions of IPF patients and TGFα transgenic mice (Moeller et al., 2009; Madala et al., 2014b). We hypothesized that increased fibrocyte survival would explain their increased accumulation in severe fibrotic lung disease. We isolated fibrocytes from the lung cultures of control and TGFα transgenic mice on Dox for 4 weeks and cultured in the presence of caspase 3/7 substrate to quantify caspase 3/7 activity. In support of our hypothesis, fibrocytes isolated from the fibrotic lung lesions of TGFα transgenic mice were more resistant to apoptosis than fibrocytes isolated from normal lungs (Figure 3A). To test the Fas-mediated apoptosis of fibrocytes, we treated them with anti-Fas antibody and quantified the apoptotic cells. We found decreased susceptibility in fibrocytes isolated from fibrotic lesions rather than normal lungs (Figure 3B). To determine whether nintedanib induces apoptosis in fibrocytes, we measured the cleaved caspase 3/7 activity in fibrocytes from the fibrotic lungs of IPF patients and TGFα transgenic mice. Cleaved caspase 3/7 activity is significantly greater in the nintedanib-treated fibrocytes of IPF (Figure 3C) and TGFα mice (Figure 3D) compared to vehicle treated.

### Overlap in the Expression of Apoptosis-Linked Genes Between the IPF and TGFα Model

**FIGURE 4:**
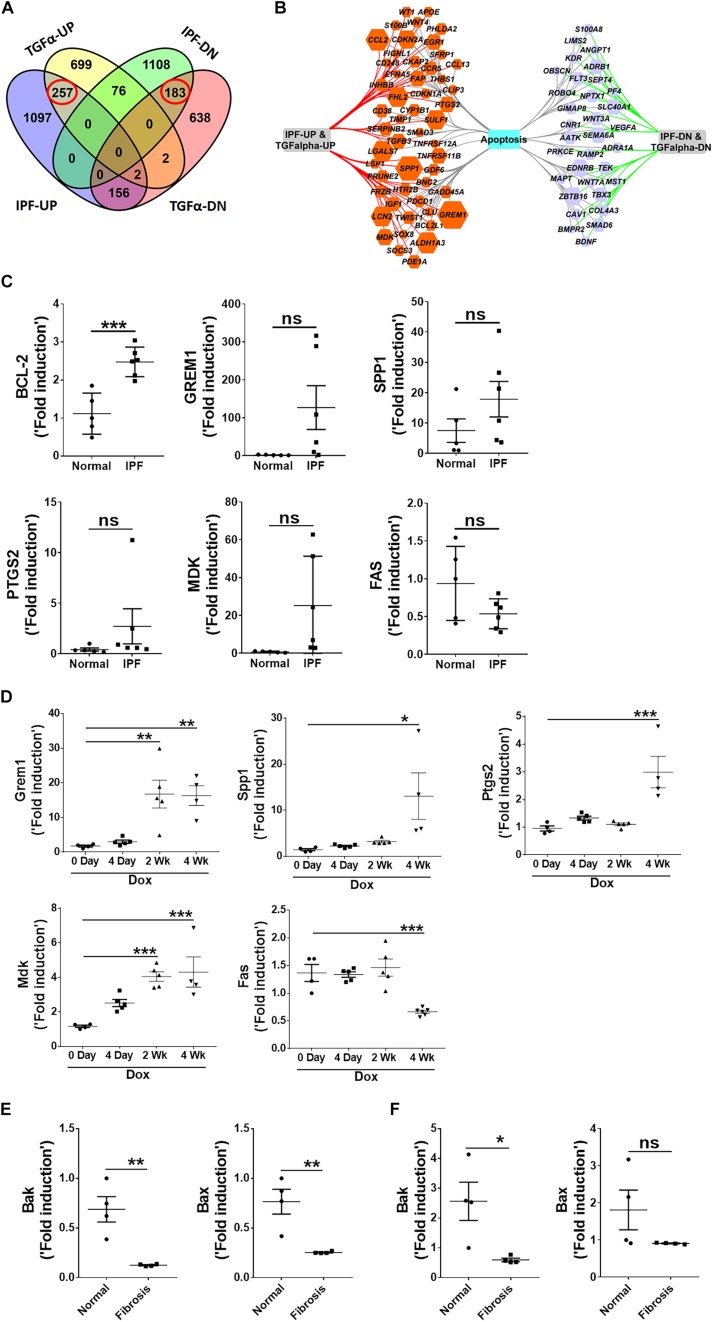
Apoptosis-linked genes are similarly dysregulated in IPF and the mouse model of TGFα-induced pulmonary fibrosis. **(A)** Venn diagram shows differentially expressed genes in IPF lungs and the TGFα mouse model. The dashed box indicates genes that are upregulated (257 genes) or downregulated (183) in IPF and the TGFα mice on Dox for 3 weeks. **(B)** The apoptosis-linked genes that are enriched and similarly dysregulated in IPF and the TGFα mice on Dox for 3 weeks are illustrated using Cytoscape. The upregulated genes are in the orange hexagons, and the downregulated genes are in the purple hexagons. Hexagon size is proportional to fold-change of gene expression in IPF. **(C)** Quantification of BCL-2, GREM1, SPP1, PTGS2, MDK, and FAS gene transcripts by RT-PCR in IPF lungs (*n* = 5–6). Unpaired Student’s *t*-test was used to measure the statistical significance between groups. **(D)** Quantification of Grem1, Spp1, Ptgs2, Mdk, and Fas transcripts by RT-PCR in the lungs of TGFα mice on Dox at 0, 4 days, 2 weeks, and 4 weeks (*n* = 4–6). One-way ANOVA with Sidak’s multiple comparisons test was used to measure a significant difference. **(E)** Quantification of Bak and Bax transcripts in lung-resident myofibroblasts (CD45^–^Col1^+^) isolated from lung stromal cell cultures from normal and TGFα mice on Dox for 4 weeks (*n* = 4). **(F)** Quantification of Bak and Bax transcripts in lung fibrocytes (CD45^+^Col1^+^) isolated from lung stromal cell cultures from normal and TGFα mice on Dox for 4 weeks (*n* = 4). All data are presented as mean ± SEM. *P < 0.05, **P < 0.005 ***P < 0.0005; ns, not significant.

To demonstrate the relevance of the TGFα transgenic mouse model in assessing the role of the mesenchymal cell survival phenotype in IPF pathogenesis, we performed a comparative expression analysis of the apoptosis-linked genes in the lungs of IPF patients and TGFα mice. Our previous studies demonstrated that after 3 weeks on Dox, TGFα transgenic mice develop fibrotic lesions, and the lung transcripts associated with fibroblast activation changes significantly (Sontake et al., 2017). Here, we performed an enrichment analysis of the gene transcripts that positively correlated with data sets for both the IPF (DePianto et al., 2015) and TGFα mice on Dox for 3 weeks (Sontake et al., 2015). Figure 4A shows overlap in the expression of dysregulated transcripts of pulmonary fibrosis. In particular, Venn analysis showed 257 and 183 gene transcripts that were similarly up- or down-regulated between IPF and TGFα mice on Dox for 3 weeks. When we performed an enrichment analysis using this overlapping gene sets, we found an enrichment for apoptosis-linked genes (Figure 4B).

To validate this finding, we used RT-PCR to analyze the expression of apoptosis-linked genes in RNA isolated from normal and IPF lungs. We observed increased expression of anti-apoptotic genes, such as Bcl2, Grem1, Spp1, Ptgs2, and Mdk, and downregulation of pro-apoptotic genes such as FAS, in the IPF lungs (Figure 4C). We also analyzed the expression of apoptosis-linked genes in the lungs of TGFα transgenic mice on Dox and observed a progressive increase in transcripts of Grem1, Spp1, Ptgs2, and Mdk during TGFα-induced pulmonary fibrosis, while Fas expression was significantly downregulated in TGFα mice on Dox for 4 weeks (Figure 4D).

To determine whether pro-apoptotic gene expression is dysregulated in mesenchymal cells during TGFα-induced pulmonary fibrosis, we used RT-PCR to quantify pro-apoptotic gene expression in lung-resident myofibroblasts and fibrocytes isolated from lungs of normal and TGFα mice on Dox for 4 weeks. We observed decrease in the expression of pro-apoptotic genes, such as Bak and Bax, in both lung-resident myofibroblasts and fibrocytes isolated from the fibrotic lungs (Figures 4E,F).

### Nintedanib Induces Pro-apoptotic Gene Expression in Both Fibrocytes and Lung-Resident Myofibroblasts

**FIGURE 5:**
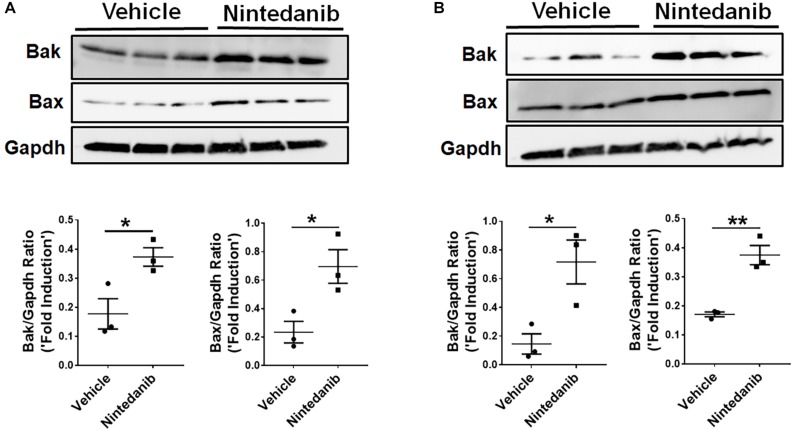
Nintedanib induces pro-apoptotic gene expression in mesenchymal cells of TGFα fibrotic mice. Primary lung-resident myofibroblasts (CD45^–^Col1^+^) and fibrocytes (CD45^+^Col1^+^) were isolated from lung stromal cell cultures of TGFα mice on Dox for 4 weeks by negative selection using anti-CD45 magnetic beads. **(A)** Immunoblot analysis of Bak, Bax, and Gapdh in fibrotic lung-resident myofibroblasts treated with vehicle or nintedanib (1 μM) for 72 h. For quantification, Bak and Bax were normalized to loading control Gapdh. **(B)** Immunoblot analysis of Bak, Bax, and Gapdh in fibrotic lung fibrocytes treated with vehicle or nintedanib (1 μM) for 72 h. For quantification, Bak and Bax were normalized to loading control Gapdh. Unpaired Student’s *t*-test was used to measure the statistical significance between groups. All data are presented as mean ± SEM (*n* = 3). *P < 0.05, **P < 0.005.

To investigate exactly how nintedanib induces apoptosis, we measured changes in pro-apoptotic gene expression in lung-resident myofibroblasts and fibrocytes treated with either vehicle or nintedanib. Nintedanib treatment resulted in upregulation of pro-apoptotic genes, such as Bak and Bax, in lung-resident myofibroblasts (Figure 5A) and fibrocytes (Figure 5B) isolated from fibrotic lung lesions of TGFα mice on Dox for 4 weeks.

### *In vivo* Nintedanib Treatment Leads to Increased Apoptosis and Reduced Proliferation and ECM Production in Mesenchymal Cells

**FIGURE 6:**
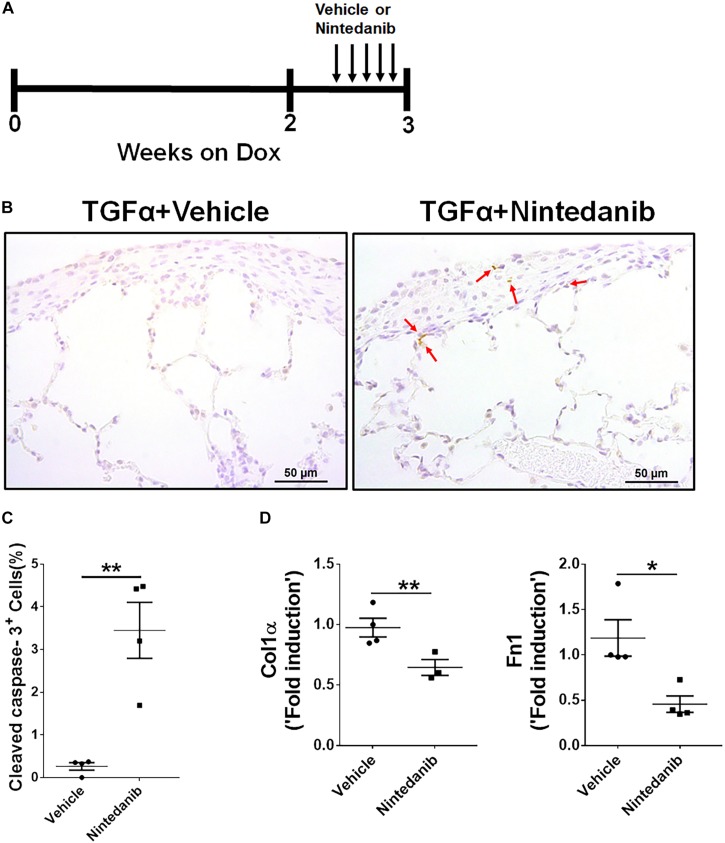
Nintedanib induces apoptosis and decreases ECM production during TGFα-induced pulmonary fibrosis. **(A)** Schematic illustration of *in vivo* nintedanib therapy protocol. TGFα mice on doxycycline (Dox) for 3 weeks were treated with vehicle or nintedanib (60 mg/kg) once a day for the last 5 days on Dox. **(B)** Immunostaining of lung sections from vehicle- or nintedanib-treated TGFα mice using active caspase-3 Ab. Images are representative of the lung subpleural region (*n* = 4/group). Scale bars: 50 μm. **(C)** Quantification of percent active caspase-3^+^ cells in subpleural regions from vehicle- or nintedanib-treated groups (*n* = 4/group; 5 representative images per animal). **(D)** Quantification of Col1α and Fn1 gene transcripts by RT-PCR in the lungs of vehicle- or nintedanib-treated TGFα mice (*n* = 4/group). All data are presented as mean ± SEM. Unpaired Student’s *t*-test was used to measure the statistical significance between groups. *P < 0.05, **P < 0.005.

To further establish the proapoptotic and anti-fibrotic effects of nintedanib *in vivo*, TGFα transgenic mice were fed on Dox-containing food for 3 weeks and the last 5 days were treated with either vehicle or 60 mg/kg of nintedanib (Figure 6A). We then performed active caspase-3 immunostaining in paraffin lung sections and found significantly increased active caspase-3-positive cells in the fibrotic lesions from the nintedanib-treated mice (Figures 6B,C), which also expressed significantly fewer ECM gene transcripts, such as collagen 1α and fibronectin1 (Figure 6D), than the vehicle-treated TGFα mice.

**FIGURE 7:**
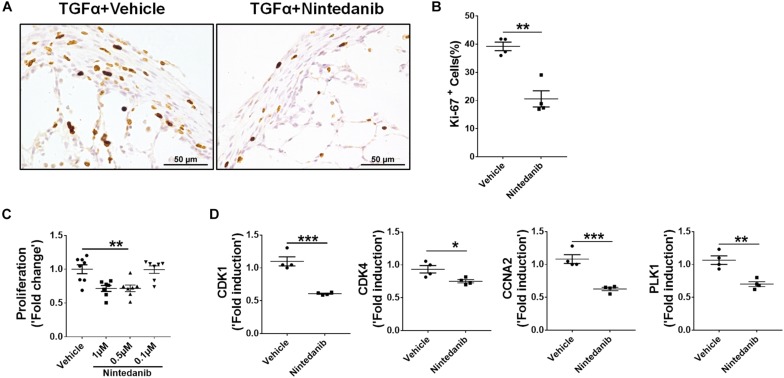
Nintedanib attenuates fibroproliferation during TGFα-induced pulmonary fibrosis. **(A)** Immunostaining of lung sections from TGFα mice treated with vehicle or nintedanib (60 mg/kg) using Ki-67 Ab. Images are representative of lung subpleura (*n* = 4/group). Scale bars: 50 μm. **(B)** Quantification of percent Ki-67^+^ cells in subpleura regions from vehicle- or nintedanib-treated groups (*n* = 4/group; 5 representative images per animal). Unpaired Student’s *t*-test was used to measure the statistical significance between groups. **(C)** Quantification of proliferation using the BrdU incorporation assay in primary lung-resident fibroblasts isolated from stromal cell cultures of TGFα mice on doxycycline (Dox) for 2 weeks. Fibroblasts were treated with the indicated doses of nintedanib for a total of 48 h. Fold-change was calculated relative to the vehicle-treated group. One-way ANOVA with Sidak’s multiple comparisons test was used to measure a significant difference. **(D)** Quantification of CDK1, CDK4, CCNA2, and PLK1 gene transcripts by RT-PCR in the primary lung-resident fibroblasts isolated from human IPF lung cultures and treated with vehicle or nintedanib (1 μM) for 16 h (*n* = 4). Unpaired Student’s *t*-test was used to measure the statistical significance between groups. All data are presented as mean ± SEM. *P < 0.05, **P < 0.005, ***P < 0.0005.

To determine whether the anti-fibrotic effects of nintedanib are in part also due to reduced proliferation, we performed Ki-67 immunostaining to measure total cell proliferation in paraffin lung sections from TGFα mice treated with nintedanib or vehicle. The nintedanib-treated mice had significantly fewer Ki-67-positive cells (Figures 7A,B). After isolating and culturing primary lung-resident fibroblasts from TGFα mice on Dox for 2 weeks in the presence or absence of nintedanib, we observed a dose-dependent effect of nintedanib in inhibiting BrdU incorporation (Figure 7C). Moreover, the expression of genes associated with cell proliferation, such as CDK1, CDK4, CCNA2, and PLK1, was significantly diminished in the lung-resident fibroblasts of IPF patients treated with nintedanib as compared to vehicle (Figure 7D). Together, these studies demonstrate that nintedanib prevented the survival, proliferation, and ECM production of mesenchymal cells *in vivo*.

### Nintedanib Therapy Attenuates the Progression of Established Pulmonary Fibrosis

**FIGURE 8:**
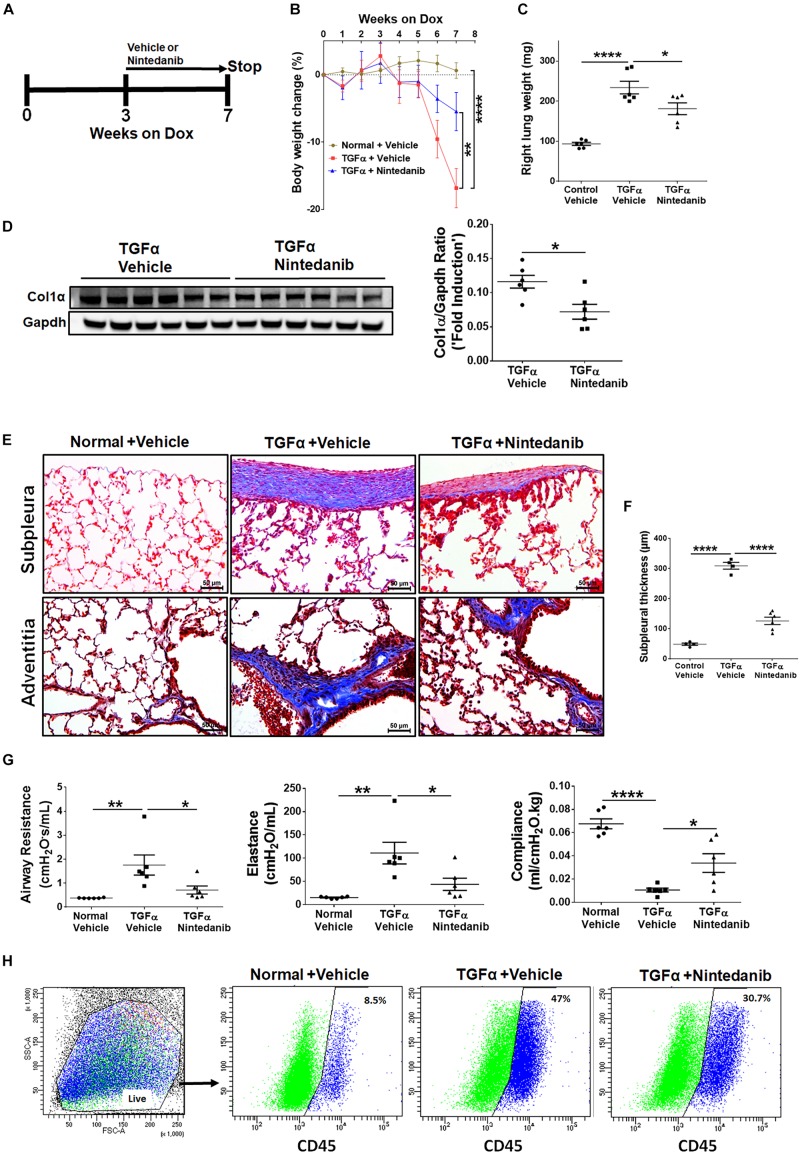
Nintedanib therapy attenuates established and ongoing pulmonary fibrosis *in vivo*. **(A)** Schematic representation of nintedanib treatment protocol. Control mice and TGFα mice were placed on Dox for 3 weeks then treated with vehicle or nintedanib (60 mg/kg; once a day) for 4 weeks while continued on Dox for a total of 7 weeks (*n* = 6/group). **(B)** The percent body weight change in mice treated with vehicle and nintedanib. **(C)** Quantitation of the right lung weight of mice in all groups treated with vehicle and nintedanib. **(D)** Quantitation of collagen 1α by western blot in lung lysates prepared from TGFα mice treated with vehicle or nintedanib (*n* = 6/group). **(E)** Images of Masson’s trichrome stained lung sections from all the groups. *Top*, subpleural regions of the lung; *bottom*, adventitia. Scale bar, 50 μm. Images are representative of each group (*n* = 5–6/ group). **(F)** Subpleural thickness was measured in the lung sections of all mice stained with Masson’s trichrome. **(G)** The lung function changes in all groups of mice treated with vehicle and nintedanib. **(H)** The representative flow cytometric analysis plots showing the percentage of CD45+ve fibrocytes in the total lung mesenchymal cells in mice treated with vehicle and nintedanib. Statistical significance was measured using one-way ANNOVA with Sidak’s multiple comparison test. *p < 0.05, **p < 0.005, ****p < 0.00005.

To determine whether long-term nintedanib therapy attenuates the progression of established fibrosis, following 3 weeks of Dox treatment when fibrosis is already established (Sontake et al., 2017), TGFα mice were treated with nintedanib while remaining on Dox for an additional 4 weeks (Figure 8A). Non-TGFα and TGFα control mice were treated with vehicle while remaining on Dox for an additional 4 weeks. Body weights of TGFα mice treated with vehicle decreased to 20% from baseline following 7 weeks of Dox (Figure 8B). Nintedanib treated TGFα mice at the beginning of week 5 decelerated body weight loss compared to vehicle-treated TGFα mice, with body weights remaining lower than vehicle treated non-TGFα control mice. Importantly, the total lung weight was significantly attenuated in TGFα mice treated with nintedanib compared to TGFα mice on Dox and vehicle treated for 7 weeks (Figure 8C). We observed a decrease in collagen deposition in the lungs of nintedanib treated compared vehicle treated TGFα mice as assessed by both western blot analysis of collagen 1α in lung lysates (Figure 8D) and Masson’s trichrome staining of lung sections (Figure 8E). We also observed a marked decrease in subpleural thickening with modest changes in perivascular and peribronchial fibrosis in nintedanib treated compared with vehicle treated TGFα mice (Figure 8F). TGFα mice treated with Dox develop severe fibrosis with significant changes in the lung mechanics compared with non-TGFα control mice on Dox for 7 weeks. Nintedanib therapy in TGFα mice has triggered decrease in lung resistance and elastance and increase in lung compliance when compared with vehicle treated TGFα mice (Figure 8G). Flow cytometric analysis of the absolute number of fibrocytes demonstrated a decrease in fibrocyte population in the lungs of TGFα mice administered with nintedanib compared to vehicle (Figure 8H and Table 3). Together, these studies demonstrate that nintedanib therapy at the time of established fibrosis modulates the progression of fibrotic disease in TGFα mice based on physiologic and histologic parameters.

**TABLE 3:** Flow cytometric analysis of an absolute number of fibrocytes.

**Fibrocytes**	**Normal + Vehicle**	**TGFα + Vehicle**	**TGFα + Nintedanib**
Absolute number	0.34 ± 0.04	2.87 ± 0.98	1.55 ± 0.36
(million/mL)			

*An absolute number of fibrocytes was measured by flow cytometry in lung stromal cell cultures of control and TGFα mice received either vehicle or nintedanib. Data are mean ± SE; *n* = 3/group.*

## Discussion

IPF is a progressive fibrotic lung disease characterized by the continuous accumulation of various mesenchymal cells that produce excessive amounts of ECM to form irreversible scar tissue in the lung. Based on transcriptome analysis of survival pathways, we propose that the IPF and TGFα models mediate the development of apoptosis resistance in fibrocytes and myofibroblasts through largely similar mechanisms to cause continuous expansion of fibrotic lesions in pulmonary fibrosis pathogenesis.

Fibrocytes are elevated in fibrotic lung lesions in several chronic lung diseases, including IPF (Moeller et al., 2009). Our previous findings suggest that they accumulate in these lesions rather than in normal-looking areas of the lung during TGFα-induced pulmonary fibrosis (Madala et al., 2014b). Our new results strongly support the idea that fibrocytes (CD45^+^Col1^+^) accumulate in fibrotic lesions because they become resistant to apoptosis. By demonstrating that fibrocytes and lung-resident myofibroblasts express low levels of pro-apoptotic proteins, including Bak and Bax, our results support the premise that they develop resistance to Fas-mediated apoptosis, further evidenced by their reduced cleaved caspase-3/7 activation when treated with Fas-activating antibody. Increasing evidence indicates that direct contribution of fibrocytes to the myofibroblast pool is limited, but they secrete several paracrine factors that induce the activation of lung-resident fibroblasts; specifically, fibroproliferation and myofibroblast transformation in the pathogenesis of pulmonary fibrosis (Hashimoto et al., 2004; Madala et al., 2014b; Sontake et al., 2015; Ashley et al., 2017). Therefore, targeting both fibrocytes and lung-resident fibroblasts is necessary for finding strategies to attenuate established pulmonary fibrosis.

Here, we used comparative expression analysis to identify apoptosis-linked genes, including midikine, gremlin1, Spp1,Ptgs2, Bak, Bax, Bcl2, and Fas, in IPF and a mouse model of TGFα-induced pulmonary fibrosis and validated changes in their expression by RT-PCR and found a significant overlap. Among upregulated genes, midikine, Spp1, and gremlin1 were shown to function as positive regulators in IPF and during bleomycin-induced pulmonary fibrosis (Pardo et al., 2005; Koli et al., 2006; Misa et al., 2017). Studies using cancer cell lines further support the hypothesis that increased expression of these genes increases the survival of mesenchymal cells, suggesting their inhibition as an intervention strategy (Tamminen et al., 2013; Muller et al., 2014; Saleh et al., 2016; Xiang et al., 2017). In particular, inhibiting the expression of gremlin-1 was sufficient to induce paclitaxel-mediated apoptosis in a mesothelioma tumor cell line (Tamminen et al., 2013). Gremlin 1 also inhibits BMP4, which has been shown to induce apoptosis in corneal fibroblasts (Mohan et al., 1998).

The balance between pro- and anti-apoptotic regulators may determine the cell fate. Here, our data suggest that several pro-apoptotic gene transcripts, including Bak, Bax, and Fas, are downregulated in the fibrotic lungs of IPF and TGFα mice. Note that Bak and Bax are downregulated in both fibrocytes and myofibroblasts isolated from the lungs of TGFα mice. In support, the survival of mouse embryonic fibroblasts (MEFs) isolated from Bax and Bak-null mice is enhanced during bleomycin-induced cell death (Lee et al., 2005). Similarly, transcript levels of Fas receptor, which initiates extrinsic apoptotic cell death upon activation by the Fas ligand, are low in murine fibrotic lungs, consistent with previous findings (Dodi et al., 2018). Furthermore, we observed resistance to Fas-mediated cell death in both fibrocytes and lung-resident myofibroblasts. Collectively, our results suggest the involvement of both intrinsic and extrinsic apoptotic pathway regulators in mesenchymal cell survival in the pathogenesis of pulmonary fibrosis. Future studies using transgenic manipulation and adoptive transfer or depletion of fibrocytes will identify the molecular regulators of their survival and their role at various stages of pulmonary fibrosis.

Although previous studies identified anti-fibrotic effects of nintedanib in multiple models of pulmonary fibrosis, we observed distinct effects of nintedanib in attenuating established and ongoing pulmonary fibrosis in TGFα mice. In particular, we show that nintedanib increases apoptotic clearance of fibrocytes and lung-resident myofibroblasts to attenuate the progression of TGFα-induced pulmonary fibrosis. In a recent study, nintedanib was shown to induce autophagy in fibroblasts isolated from IPF lungs (Rangarajan et al., 2016). In addition, nintedanib can inhibit fibrocyte migration, thereby reducing the number of fibrocytes in the lung during bleomycin-induced pulmonary fibrosis (Sato et al., 2017). Consistent with previous reports, our

*in vitro* and *in vivo* studies confirm that nintedanib therapy attenuates proliferation and the expression of proliferation-associated genes. Our new findings suggest that nintedanib inhibits the survival of both fibrocytes and lung-resident myofibroblasts by inducing pro-apoptotic gene expression during severe fibrotic lung disease. However, the above effects may not be limited to mesenchymal cells as other lung cells such as epithelial cells are susceptible to nintedanib, which may have beneficial or detrimental effects on normal regenerative processes in injured lungs. In support, a recent study by Lehmann et al. (2018) show that nintedanib treatment stabilizes the expression of distal epithelial cell markers such as SP-C in murine and human lung tissue cultures. Therefore, future studies are warranted to understand the dose dependent effects of nintedanib on multiple lung cells including mesenchymal, epithelial, endothelial and immune cells involved in anti-fibrotic and repair processes. These studies would be critical to develop more efficacious therapies against severe fibrotic lung diseases. Nevertheless, clinical development of effective combination therapies that can reverse established and ongoing fibrosis has been hindered by the lack of knowledge on mechanism of action and also undesired side effects associated with nintedanib therapy. Modulating the activity of fibroblasts might serve as a valuable approach to treat severe fibrotic lung disease, in particular activity of multiple growth factors. Our previous studies demonstrate upregulation of multiple growth factors including TGFβ, IL-6, IL-13, amphiregulin and epiregulin in distinct mesenchymal cell subsets such as fibrocytes, lung resident fibroblasts and WT1-positive myofibroblasts during TGFα-induced pulmonary fibrosis (Madala et al., 2014c; Sontake et al., 2015, 2018). TGFα was found upregulated in multiple fibrotic lung diseases including IPF and cystic fibrosis (Baughman et al., 1999; Hardie et al., 1999). A number of reports demonstrates that inhibiting multiple signaling pathways markedly prevent TGFα-induced pulmonary fibrosis (Madala et al., 2014a, 2016a, b). However, incomplete resolution of lung fibrosis with nintedanib therapy and other inhibitors is not unique to the TGFα model. In a recent study using SKG mice with joint disease by Redente et al. (2018) have found that nintedanib therapy attenuates collagen deposition but did not alter lung compliance. The variability in the effects of nintedanib likely reflect differences in the relative contribution of multiple pro-fibrotic proteins and pathways in mediating fibrogenesis amongst the fibrosis models and strains of mice studied.

**FIGURE 9:**
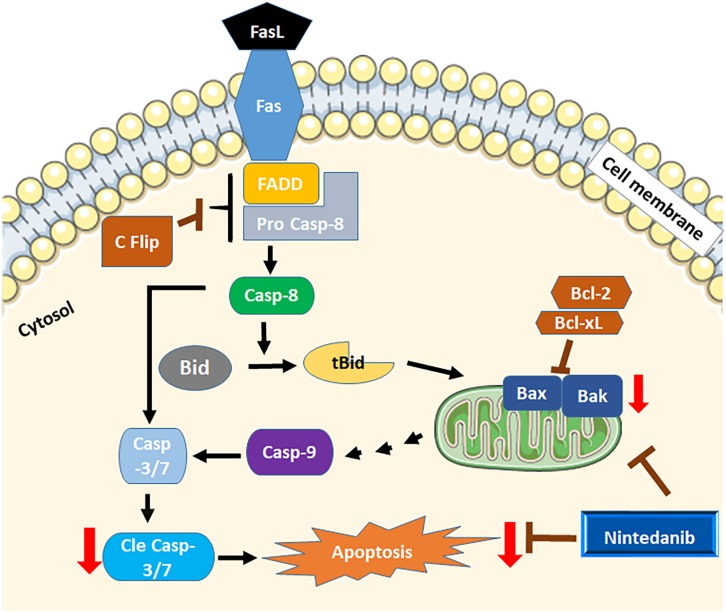
Schematicview of apoptosis by nintedanib. Both intrinsic and extrinsic apoptotic regulators are dysregulated in mesenchymal cells of fibrotic lungs. Nintedanib induces apoptosis by increasing the expression of pro-apoptotic genes Bak and Bax.

In conclusion, we identified a role for resistance to apoptosis in regulating fibrocyte accumulation in the pathogenesis of pulmonary fibrosis. Our new results show that nintedanib induces apoptosis in both fibrocytes and lung resident myofibroblasts. This increase in apoptotic clearance is associated with increased expression of the pro-apoptotic genes Bak and Bax by nintedanib (Figure 9). Our *in vitro* studies using primary cells and *in vivo* study using a mouse model of TGFα-induced pulmonary fibrosis demonstrates that nintedanib therapy is effective to attenuate fibroblast activation including fibroproliferation, resistance to apoptosis and ECM production in the mature fibrotic lung lesions. Our new findings highlight the relevance of using a mouse model of TGFα-induced pulmonary fibrosis to study fibroblast activation in the pathogenesis of fibrotic lung disease. Future studies employing this model either alone or combined with other chronic fibrotic lung disease phenotypes that do not resolve will be useful to examine manipulation of lung regeneration and to test the efficacy of nintedanib against IPF in combination with other drugs.

## Data Availability

Publicly available datasets were analyzed in this study. This data can be found here: https://www.ncbi.nlm.nih.gov/geo/query/acc.cgi?acc=GSE53845.

## Ethics Statement

This study was carried out in accordance with protocols approved by the Institutional Animal Care and Use Committee of the Cincinnati Children’s Hospital Research Foundation.

## Author Contributions

RK and SM conceived and designed the research. RK, SM, AJ, and GR performed the experiments and wrote the manuscript.

## Disclaimer

None of the authors has a financial relationship with a commercial entity that has an interest in the subject of this manuscript.

## Conflict of Interest Statement

The authors declare that the research was conducted in the absence of any commercial or financial relationships that could be construed as a potential conflict of interest.
